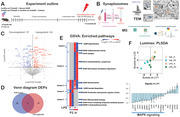# Neuroinflammatory Stress Exerts Distinct Proteomic Effects on Soma, Synapses and Mitochondria in Excitatory Neurons

**DOI:** 10.1002/alz.090723

**Published:** 2025-01-03

**Authors:** Claudia Espinosa‐Garcia, Upasna Srivastava, Prateek Kumar, Brendan R Tobin, Hailian Xiao, Lihong Cheng, Pritha Bagchi, Duc M Duong, Nicholas T Seyfried, Victor Faundez, Levi B Wood, Srikant Rangaraju

**Affiliations:** ^1^ Yale University School of Medicine, New Haven, CT USA; ^2^ Parker H Petit Institute for Bioengineering and Biosciences, Georgia Institute of Technology, Atlanta, GA USA; ^3^ Georgia Institute of Technology, Atlanta, GA USA; ^4^ Emory University Center for Neurodegenerative Disease, Atlanta, GA USA; ^5^ Emory University School of Medicine, Atlanta, GA USA; ^6^ Center for Neurodegenerative Disease, Atlanta, GA USA

## Abstract

**Background:**

Neuroinflammation plays a critical role in Alzheimer’s disease pathogenesis. Neurons are anatomically divided in subcellular compartments (axons, soma, and synapses), which may be distinctly impacted by neuroinflammation. This study aims to examine cellular compartment‐specific proteomic signatures in excitatory neurons following a systemic neuroinflammatory stress.

**Method:**

We used our innovative CIBOP (cell type‐specific *in vivo* biotinylation of proteins) approach to selectively label Camk2a excitatory neuron proteomes *in vivo*. Neuron‐CIBOP transgenic mice and their littermate controls were treated with lipopolysaccharide (LPS, [500 µg/kg, i.p.]) during 4 consecutive days, which induces robust microglial activation and sickness behavior. After euthanasia, brains were quickly removed, and crude synaptosomal fractions (P2 fractions) were prepared by differential centrifugation. Neuron‐specific biotinylated proteins were then enriched and analyzed by label‐free quantitative mass spectrometry (MS) to identify differentially‐enriched proteins (DEPs) and biological pathways (by gene set variation analysis/GSVA). Neuron‐derived biotinylated key cellular signaling pathways (MAPK and Akt/mTOR) were directly measured by Luminex in homogenates and P2 fractions (Fig. 1A).

**Result:**

Electron micrographs validated the subcellular composition of the P2 fractions showing synaptosomes containing synaptic vesicles and mitochondria (Fig. 1B). MS studies confirmed that neuronal homogenates were enriched in microtubule and cytoskeleton‐related proteins, while P2 fractions were enriched in mitochondria and synapse‐related proteins (Fig. 1C). Interestingly, LPS induced unique compartment‐specific proteomic effects, P2 fraction has 52 unique DEPs, while homogenate has 57 DEPs, and only 2 DEPs overlapped (Fig. 1D). Neuronal homogenate proteomes showed upregulation of detoxification and oxidoreductase activity, while a reduced neuron‐synapse, somatodendritic compartment, and cytoskeleton organization. In P2 fraction proteomes, LPS upregulated mitochondrial envelope formation and metabolic activity, including purine containing compound metabolic process, but downregulated nucleoside triphosphate regulator activity. Increased aerobic respiration and mitochondria response overlapped among compartments (Fig. 1E). We also observed LPS‐induced decrease in MAPK signaling specifically in the P2 fraction, not evident at the level of whole neurons, nor at the bulk brain tissue level (Fig. 1F).

**Conclusion:**

Our neuron and synaptosome‐enriched proteomics approach revealed unique molecular and signaling effects of neuroinflammation that may preferentially impact the synapses of excitatory neurons.